# The role of structured inpatient lipid protocols in optimizing non-statin lipid lowering therapy: a review and single-center experience

**DOI:** 10.3389/fcvm.2024.1284562

**Published:** 2024-01-25

**Authors:** Chad Gier, Ian Gilchrist, Matthew Fordham, Luke Riordan, Ella Milchan, Nidhi Patel, Azad Mojahedi, Sahana Choudhury, Tara Kits, Regina Cohen, Joseph Doughtery, John P. Reilly, Andreas Kalogeropoulos, Tahmid Rahman, On Chen

**Affiliations:** ^1^ Stony Brook Heart Institute, Stony Brook University Hospital, Stony Brook, NY, United States; ^2^ Stony Brook Renaissance School of Medicine, Stony Brook, NY, United States

**Keywords:** hyperlipidemia, quality improvement, ASCVD, lipid lower medications, LDL—Cholesterol

## Abstract

Dyslipidemia is a leading contributor to atherosclerotic cardiovascular disease (ASCVD). There has been a significant improvement in the treatment of dyslipidemia in the past 10 years with the development of new pharmacotherapies. The intent of this review is help enhance clinicians understanding of non-statin lipid lowering therapies in accordance with the 2022 American College of Cardiology Expert Consensus Clinical Decision Pathway on the Role of Non-statin Therapies for LDL-Cholesterol Lowering. We also present a single-center experience implementing a systematic inpatient protocol for lipid lowering therapy for secondary prevention of ASCVD.

## Introduction

Atherosclerotic cardiovascular disease (ASCVD) is one of the leading causes of mortality in the United States. Dyslipidemia is a major contributor to ASCVD, and aggressive cholesterol lowering is a cornerstone of secondary prevention. Despite the recognition of the importance of cholesterol reduction, studies have demonstrated that many patients are receiving suboptimal therapy (1, 2).

For patients at clinical ASCVD a systematic and aggressive combination therapy approach may be necessary to ensure that patients receive optimal lipid-lowering therapy (LLT) to reduce the risk of recurrent cardiovascular events.

In this article, we present our institution's structured inpatient lipid protocol as an example of how structured protocols may potentially improve prescribing of appropriate combination LLT and enhance the achievement of LDL-C thresholds. We also summarize the non-statin LLT available in the United States and how they fit into the recently published 2022 ACC expert consensus decision pathway on the role of non-statin therapies for low-density lipoprotein cholesterol (LDL-C) lowering in the management of ASCVD risk (referred to in this paper as 2022 ACC ECDP).

## 2022 American College of Cardiology expert consensus decision pathway and “new” thresholds

The 2018 American Heart Association/American College of Cardiology guidelines recommend an LDL-C threshold of <70 mg/dl in very-high-risk patients (defined as a history of multiple major ASCVD events or 1 major ASCVD event with multiple high-risk conditions) (3). However, the 2019 European Society of Cardiology and European Atherosclerosis Society (ESC/EAS) guidelines recommend a lower LDL-C threshold of <55 mg/dl. In their guidelines, they note further absolute lowering of LDL-C lowers the risk of ASCVD (4). This evidence prompted the 2022 ACC ECDP to similarly update their recommendations to more aggressive LDL-C threshold of <55 mg/dl in patients with clinical ASCVD at very high risk (5). For patients with clinical ASCVD but not at high-risk, the threshold remains <70 mg/dl or non-HDL <100 mg/dl. In addition to changes in lipid thresholds, the 2022 ACC ECDP also emphasized the use of combination therapy, including the use of proprotein convertase subtilisin/kexin type 9 (PCSK9) as first-line treatments following statin use.

### Available non-statin lipid lowering pharmacotherapies

As a result of the identification of new therapeutic thresholds and the development of effective pharmacotherapies, the available non-statin lipid-lowering medicines have expanded dramatically during the past several years. The estimated lipid-lower effect of these medications, when added to statin therapy ranges from 15%–60%. Details of each pharmacotherapy are described below.

#### Ezetimibe

Ezetimibe acts by inhibiting the Niemann-Pick C1-like 1 (NPC1L1) protein, which reduces intestinal absorption of cholesterol and reduces enterohepatic recirculation of bile (6). Ezetimibe has been estimated to reduce LDL by 20%–25% (7) when added to maximally tolerated statin therapy.

The IMPROVE-IT trial enrolled patients with acute coronary syndrome to assess the efficacy of ezetimibe in reducing LDL-C and the risk of cardiovascular events. The IMPROVE-IT trial randomized 18,144 patients to receive either simvastatin with ezetimibe or simvastatin monotherapy. IMPROVE-IT showed the addition of ezetimibe to statin monotherapy significantly reduced a composite of cardiovascular death, major coronary events, or nonfatal stroke by 2.0% (8).

Previously, the 2018 AHA/ACC cholesterol guidelines recommended ezetimibe as the initial treatment in patients with clinical ASCVD and LDL-C >70 mg/dl on maximally tolerated statin therapy (3). However, monoclonal antibody PCSK9 inhibitors are now also recommended as a first-line non-statin treatment option and should be preferred over ezetimibe if a reduction in LDL of more than 25% is desired (5).

#### Monoclonal antibodies targeting PCSK9

Monoclonal antibodies (mAb) that inhibit PCSK9, a key intermediary that signals for the degradation of LDL receptors in the liver, have been breakthrough medications for the treatment of hypercholesteremia (6). PCSK9 inhibitors were first incorporated in the 2018 AHA/ACC cholesterol guidelines after the landmark results of the FOURIER and ODYSSEY trials. mAb PCSK9 inhibitors when added to maximally tolerated statin therapy have been estimated to reduce LDL by approximately 60% (9, 10).

The FOURIER trial was a large, randomized controlled trial that evaluated the effects of evolocumab in combination with optimized LLT (defined as atorvastatin 20 mg daily or its equivalent with or without ezetimibe) in patients with established clinical ASCVD. In the trial, evolocumab combined with LLT reduced LDL-C levels by almost 60% and showed a significant reduction in the risk of a composite of cardiovascular death, myocardial infarction, stroke, hospitalization for unstable angina, or coronary revascularization by 15%. Individually, there was a 27% decrease in the risk of myocardial infarction, 21% decrease in risk of stroke, and 22% risk in the need for coronary revascularization. There was no significant reduction in all-cause mortality or cardiovascular death (9).

Recently the FOURIER open label extension (OLE) long term outcomes were published in Circulation. The OLE followed patients for an extended period including a median of 5 years. At 12 weeks, the median LDL-C was 30 mg/dl, and 63% of patients on evolocumab had LDL-C <40 mg/dl. Patients taking evolocumab in addition to their previous LLT had 15% lower risk of a composite of cardiovascular death, myocardial infarction, stroke, or hospitalization for unstable angina/coronary revascularization. The OLE study also showed that patients in the evolocumab arm also had 23% lower risk of cardiovascular death. No significant difference in adverse events including muscle-related symptoms, new-onset diabetes, hemorrhagic stroke, and neurocognitive events was observed between evolocumab vs. placebo (11).

The ODYSSEY OUTCOMES trial evaluated longer term data on LDL-C reduction and cardiovascular outcomes in patients following acute coronary syndrome. The trial showed the addition of alirocumab significantly reduced LDL-C levels by over 62% at 4 months. In *post hoc* analysis, there was also a significant reduction in a composite of death from composite of coronary heart disease, nonfatal myocardial infarction, fatal or nonfatal ischemic stroke, or unstable angina requiring hospitalization by 15% (HR 0.85; 95% CI, 0.78–0.93; *p* < 0.001) (10, 12).

In the 2018 AHA/ACC cholesterol guidelines, mAb PCSK9 inhibitors were only recommended if a very-high-risk patient was not at goal who was already on maximally tolerated statin therapy and ezetimibe (3). In the 2022 ACC ECDP, PCSK9 mAb are recommended as a first-line non-statin therapy in patients with clinical ASCVD and especially if patient requires >25% additional lowering of LDL-C (5).

#### Small interfering ribonucleic acid (siRNA) targeting PCSK9

Inclisiran is a small interfering RNA (siRNA) drug that specifically targets the mRNA for the enzyme PCSK9 produced by the liver. Reduction in PCSK9 increases available LDL receptors which significantly decreases plasma LDL levels (6).

In a pooled analysis of three phase 3 clinical trials, inclisiran demonstrated a mean placebo-corrected reduction in low-density lipoprotein cholesterol (LDL-C) levels of 50% at 510 days (13). The effects of inclisiran on cardiovascular morbidity and mortality are currently being evaluated in two ongoing cardiovascular outcomes trials (14, 15).

One significant advantage of inclisiran is that it can be administered as a subcutaneous injection on an annual or biannual basis, which may improve patient adherence to treatment (5).

According to the 2022 ECDP, inclisiran may be a second-line treatment option given lack of outcomes data compared to mAb PCSK9 inhibitors. Specifically, recommendations are made for patients who have poor adherence to PCSK9 mAbs, adverse effects from PCSK9 mAbs, or are unable to self-inject (5).

#### Bempedoic acid

Bempedoic acid is a prodrug that inhibits adenosine triphosphate citrate lyase (ACL), which is one-step prior to HMG-CoA reductase in the cholesterol synthesis pathway. By inhibiting ACL, bempedoic acid reduces the production of cholesterol in the liver and lowers cholesterol levels in the blood (6). As a prodrug, bempedoic acid is activated only in the liver, which may reduce muscular side effects that plague statins.

The CLEAR trials were a series of large, randomized-controlled clinical trials that evaluated the effectiveness and safety of bempedoic acid in individuals with hypercholesterolemia or mixed dyslipidemia. Bempedoic acid added to maximally tolerated statin therapy lowers LDL-C by 15%–18%. In patients unable to tolerate statin therapy due to statin-associated muscle symptoms, bempedoic acid monotherapy resulted in approximately 25% reduction in LDL-C (16–18). The CLEAR Outcomes was recently presented at the 2023 ACC annual meeting, and published in the New England Journal of Medicine. The CLEAR Outcomes trial was a double-blind, randomized, placebo-controlled trial that included statin-intolerant patients deemed to be high risk for cardiovascular disease. The trial showed that bempedoic acid resulted in a 13% reduction in the risk of the primary endpoint of major adverse cardiovascular event, death from cardiovascular causes, nonfatal myocardial infarction, nonfatal stroke, or coronary revascularization when compared to placebo (19).

The 2022 ACC ECDP recommends bempedoic acid as a second-line non-statin medication, following PCSK9 inhibitors and ezetimibe, due to the lack of available outcome data at the time of publication (5).

#### Icosapent ethyl

Icosapent ethyl inhibits the enzyme delta-5 desaturase, which is involved in the synthesis of very low-density lipoprotein (VLDL) cholesterol. The inhibition of delta-5 desaturase reduces the production of VLDL cholesterol and lowers triglyceride levels in the blood (6).

The 2015 REDUCE-IT trial was a randomized, double-blind, placebo-controlled clinical trial that evaluated the effectiveness of the drug icosapent ethyl for the treatment of patients with high triglycerides and established ASCVD. The trial enrolled 8,179 patients who were assigned to receive either icosapent ethyl or a placebo. The REDUCE-IT trial showed that treatment with icosapent ethyl significantly reduced the risk of the a composite of cardiovascular death, nonfatal myocardial infarction, nonfatal stroke, coronary revascularization, or unstable angina requiring hospitalization by 25% compared to placebo (HR 0.75; 95% CI, 0.68–0.83; *p* < 0.001) (20).

Icosapent ethyl is primarily used to lower triglyceride levels and is not specifically addressed in the 2022 ACC ECDP, which primarily focuses on recommendations for optimal LDL-C lowering therapies (5). Interestingly, the benefit of icosapent ethyl appears to be independent of triglyceride reduction and may be due to other pleotropic effects which should be investigated in future studies.

#### Other specialized therapies

The 2022 ECDP also discusses evinacumab, lomitapide, however, these are reserved for patients with specific familial hypercholesterolemia syndromes. Similarly, LDL apheresis should only be considered in specialized situations, for patients under the care of lipid specialists (5).

##### Structured lipid treatment protocol and referral to advanced lipid management program

At our tertiary care academic medical center, we conducted a quality improvement study to assess the proportion of patients admitted with acute coronary syndromes (non-ST-elevation myocardial infarction or ST-elevation myocardial infarction) who underwent percutaneous coronary intervention and were prescribed LLT at discharge from 1/1/2018 to 08/20/2021. Our analysis found that 92% of these patients were prescribed statin therapy, but only 66% of them had an LDL-C level below 70 mg/dl at their subsequent follow-up appointment.

**Figure 1:**
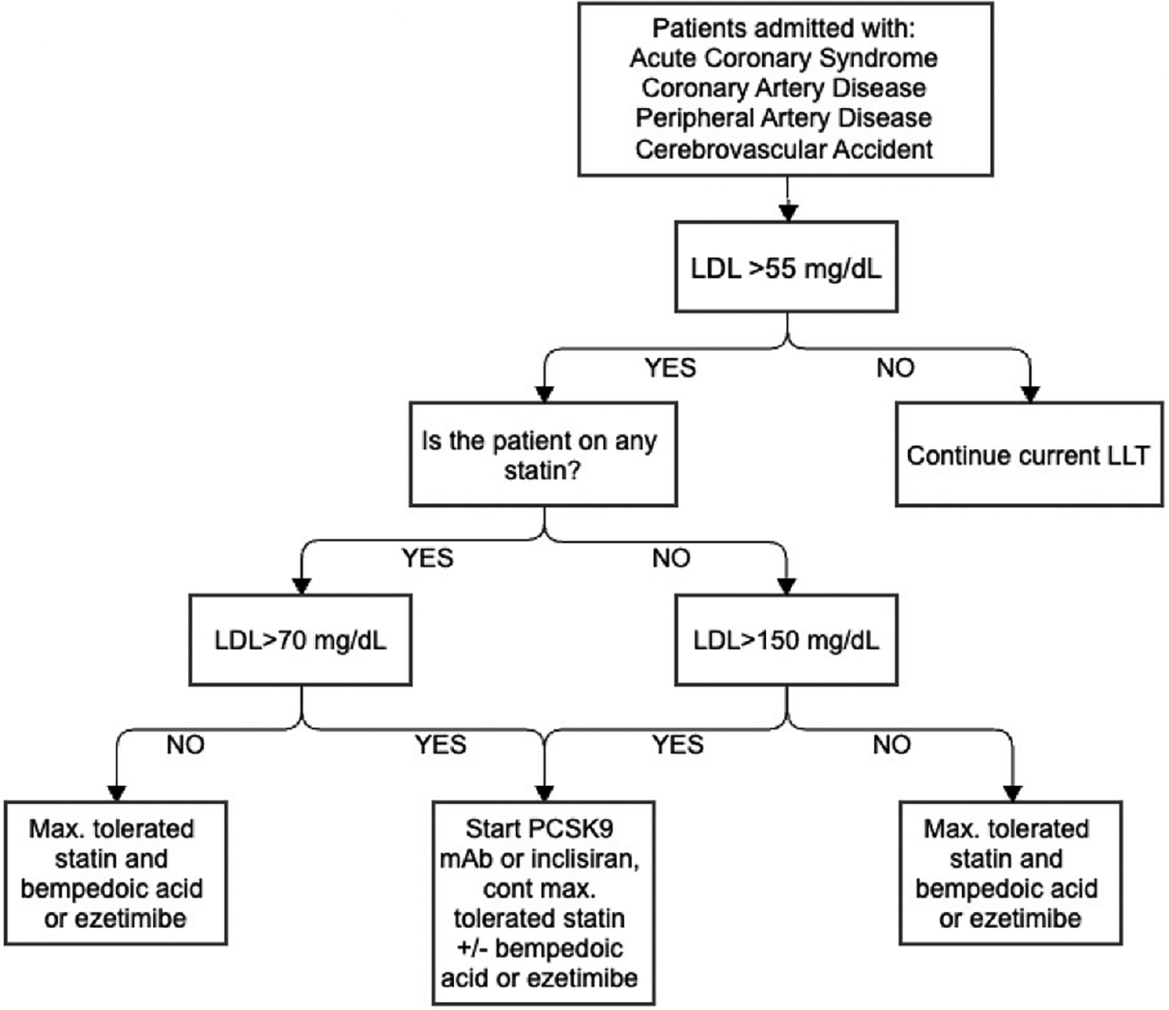
Stony Brook University Hospital inpatient lipid protocol.

Despite a higher proportion of patients meeting their LDL-C thresholds compared to the national average following an ASCVD event, a high proportion of patients did not reach their threshold LDL-C, leading us to pursue improvements in this area (21). Therefore, a structured inpatient lipid protocol was created to identify very-high-risk patients, standardize the initiation and escalation of LLT, and ensuring appropriate monitoring and follow-up. Our structured inpatient protocol is outlined in Figure 1.

When designing the protocol, we actively engaged relevant stakeholders including physicians, pharmacists, and administrators in the developmental/planning stages to ensure it was feasible and pragmatic. Second, comprehensive training regarding lipid lowering therapy was delivered to the cardiology fellows, pharmacists, nurses, and resident physicians who care for patients on the cardiology units. Third, the protocol was aligned with existing clinical workflows to ensure that it was seamlessly adopted into clinical practice.

The protocol starts by identifying patients who have not achieved the LDL-C threshold recommended by the ACC secondary prevention of ASCVD. Previously, our protocol set this threshold at less than 70 mg/dl, but it has been updated to align with the new ACC ECDP recommendation of less than 55 mg/dl for very-high-risk patients. For patients admitted with an ASCVD event, we recommend combination therapy for all patients as statin monotherapy alone is unlikely to lower their LDL to the threshold of less than 55 mg/dl. If patients' LDL is not already at goal, we add a mAb PCSK9 inhibitor or inclisiran if the patient is previously on a statin and LDL >70 mg/dl, or the patient is not on a statin and LDL >150 mg/dl. If patients' LDL is <70 mg/dl and previously on a statin or if LDL is <150 mg/dl not previously on a statin, we prescribe combination therapy including maximally tolerated statin with bempedoic acid or ezetimibe. If patients are unable to receive mAb PCSK9 inhibitor or inclisiran due to cost or insurance approval issues, we initiate additional therapy with ezetimibe or bempedoic acid.

A critical aspect of our inpatient lipid protocol is the participation of a multidisciplinary team, including nurses, care managers, physicians, and pharmacists, in daily rounds to discuss the care of patients identified by primary teams or ICD codes for intensification of LLT. This approach has been implemented for several reasons: first, to ensure that all members of the healthcare team are informed about the treatment plan for each patient; second, to ensure that patients receive appropriate therapy as soon as possible after an ASCVD event; and third, to identify any barriers to medication use, such as the need for insurance pre-approval or prohibitive cost.

**Figure 2:**
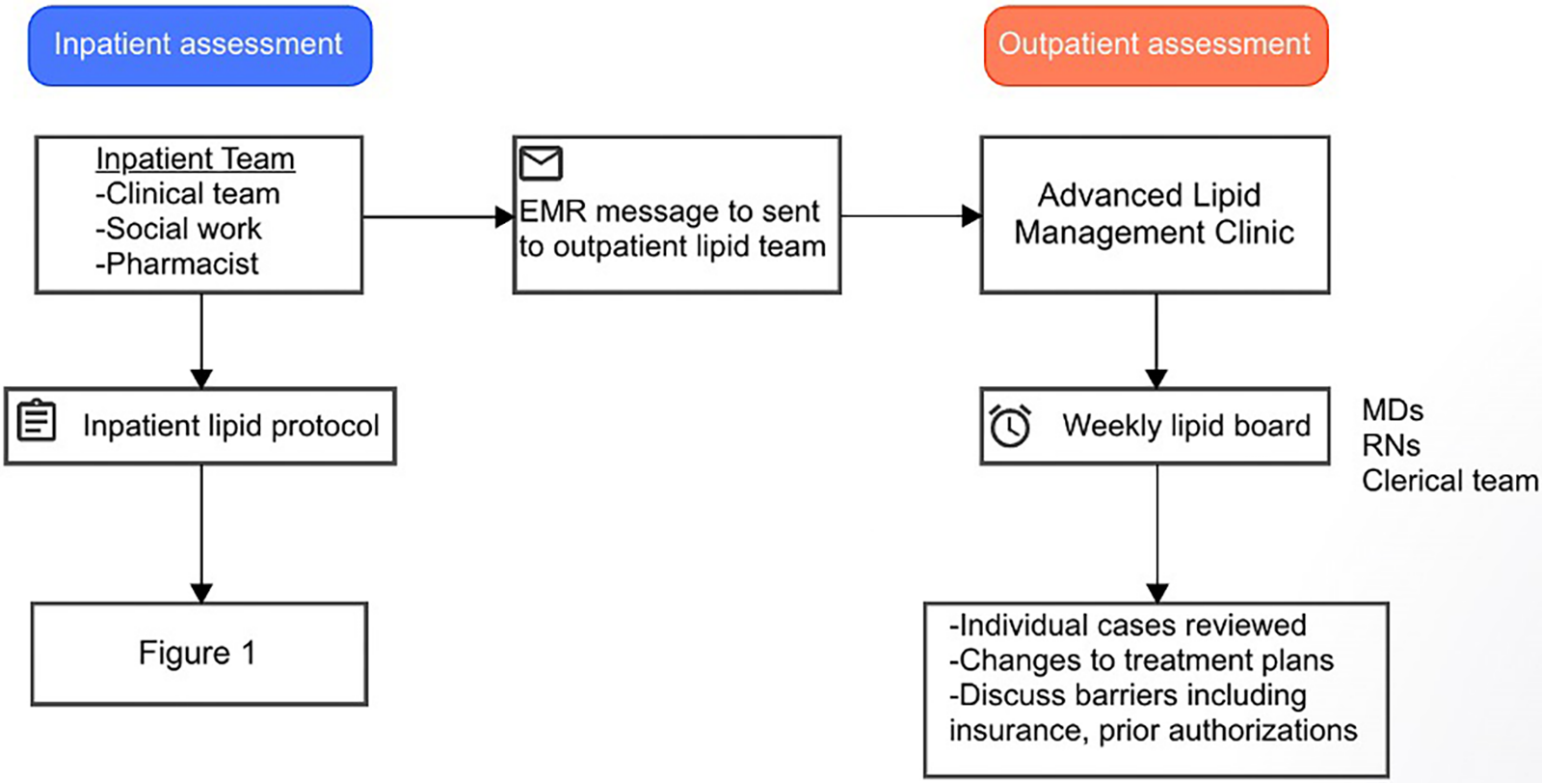
Advanced lipid management program inpatient-to-outpatient bridge.

Prior to discharge, all patients are also screened for referral to the Advanced Lipid Management Program (ALMP). Typically, patients are referred to the ALMP if they meet the criteria for a “high risk” condition including patients with LDL >190 mg/dl on maximally tolerated statin therapy, LDL >250 mg/dl without therapy, apolipoprotein B >200 mg/dl, lipoprotein (a) >150 nmol/L, triglycerides >1,000 mg/dl, LDL thresholds cannot be reached on PCSK9 inhibitor use, concern for familial hypercholesterolemia/familial chylomicronemia syndrome, or need for alternative to statin or initiation of inclisiran. If patients meet the criteria for ALMP referral, a message is sent to the outpatient lipid team and follow-up appointment is scheduled within four weeks of discharge to repeat the lipid panel and assess medication tolerance. Additionally, cases are reviewed during a weekly lipid board to discuss treatment plans and other issues such as pre-approval, cost, and refills (Figure 2). All patients who follow in the ALMP undergo lifestyle modification training including direct education, online video resources, and direct telehealth visits focused on diet and exercise with nurse practitioners.

#### Future directions

Currently, a pre-post (or before-after) study is being conducted at our institution to assess the achievement of LDL-C thresholds and clinical outcomes in patients admitted with an ASCVD events following the implementation of our inpatient lipid protocol in our electronic medical record. To continually improve the enactment of our protocol, we regularly seek input and feedback from stakeholders (physicians, pharmacists, other members of multidisciplinary team) who are directly involved in the execution of the protocol. Specifically, we seek identification of challenges, track adherence rates, and patient outcomes.

## Conclusion

The 2022 ACC expert consensus decision pathway (ECDP) on the role of non-statin therapies for LDL-C lowering recommends aggressive combination therapy to lower LDL-C in very-high-risk patients. To effectively manage very-high-risk patients and ensure that they receive appropriate lipid-lowering therapy, it may be necessary to implement a structured inpatient protocol.
